# The impact of multisensory integration deficits on speech perception in children with autism spectrum disorders

**DOI:** 10.3389/fpsyg.2014.00379

**Published:** 2014-05-21

**Authors:** Ryan A. Stevenson, Magali Segers, Susanne Ferber, Morgan D. Barense, Mark T. Wallace

**Affiliations:** ^1^Department of Psychology, University of TorontoToronto, ON, Canada; ^2^Department of Psychology, York UniversityToronto, ON, Canada; ^3^Rotman Research InstituteToronto, ON, Canada; ^4^Department of Hearing and Speech Sciences, Vanderbilt University Medical CenterNashville, TN, USA; ^5^Vanderbilt University Medical Center, Vanderbilt Brain InstituteNashville, TN, USA; ^6^Vanderbilt Kennedy CenterNashville, TN, USA; ^7^Department of Psychology, Vanderbilt UniversityNashville, TN, USA; ^8^Department of Psychiatry, Vanderbilt University Medical CenterNashville, TN, USA

**Keywords:** autism spectrum disorders (ASD), autism, multisensory integration, audiovisual, audiovisual processing, developmental disabilities, sensory perception, speech perception

Speech perception is an inherently multisensory process. When having a face-to-face conversation, a listener not only hears what a speaker is saying, but also sees the articulatory gestures that accompany those sounds. Speech signals in visual and auditory modalities provide complementary information to the listener (Kavanagh and Mattingly, 1974), and when both are perceived in unison, behavioral gains in in speech perception are observed (Sumby and Pollack, 1954). Notably, this benefit is accentuated when speech is perceived in a noisy environment (Sumby and Pollack, 1954). To achieve a behavioral gain from multisensory processing of speech, however, the auditory and visual signals must be perceptually bound into a single, unified percept. The most commonly cited effect that demonstrates perceptual binding in audiovisual speech perception is the McGurk effect (McGurk and MacDonald, 1976), where a listener *hears* a speaker utter the syllable “ba,” and *sees* the speaker utter the syllable “ga.” When these two speech signals are perceptually bound, the listener perceives the speaker as having said “da” or “tha,” syllables that are not contained in either of the unisensory signals, resulting in a perceptual binding, or integration, of the speech signals (Calvert and Thesen, 2004).

The ability to perceptually bind sensory information is notably impaired in a number of clinical populations, including those with autism spectrum disorders (ASD). ASD describes a cluster of highly prevalent developmental disabilities historically characterized by deficits in three functional domains: language and communication, social reciprocity, and the presence of restricted interests/repetitive behaviors (APA, 2000). Since its initial description, alterations in sensory processing have been described in this population (Kanner, 1943), yet these deficits were acknowledged only in the most recent edition of the DSM (APA, 2013). Impairments in multisensory perceptual binding may be particularly relevant in ASD, given that hallmark features of the disorder include difficulties in speech, communication, and social interactions. Successful speech communication is heavily reliant on binding across sensory modalities, and as such, impaired binding in individuals with ASD likely contributes to these core deficits.

Impairments in perceptual binding have not gone unstudied in ASD. In fact, one of the leading theories describing ASD, Weak Central Coherence, describes ASD as a cognitive style in which focus is selectively attuned to individual components of information to the exclusion of perceiving the larger whole; in short, losing the proverbial forest for the trees (Frith and Happé, 1994; Happé, 1999, 2005; Happé and Frith, 2006). Evidence for this has been found across a wide range of tasks. For example, individuals with ASD benefit less than individuals without ASD from context when interpreting a sentence or story (Happé, 1994; Jolliffe and Baron-Cohen, 1999), but are more accurate than individuals without ASD when focusing on explicit local details of a passage (Noens and Berckelaer-Onnes, 2005).

In the realm of sensory perception, binding deficits in ASD have been studied most extensively in the visual modality. Here too, individuals with ASD have been shown to have a strong local bias at the expense of global processing (Behrmann et al., 2006). A clear example of this is observed in response to hierarchical letters (large letters composed of smaller letters; Navon, 1977). When performing a task reliant upon the identify the gestalt of the image (the large letter) relative to the individual units (small component letters), individuals with ASD show impaired performance (Behrmann et al., 2006).

The ability of individuals with ASD to bind *across* sensory modalities has been studied to a much lesser extent, but those studies that have been conducted commonly find deficits in multisensory perceptual binding, particularly with speech signals. The majority of the research suggests that individuals with ASD perceive the McGurk illusion less often than their peers without ASD (de Gelder et al., 1991; Williams et al., 2004; Mongillo et al., 2008; Irwin et al., 2011; Bebko et al., 2014; Stevenson et al., 2014, in press; but see Iarocci and McDonald, 2006; Woynaroski et al., 2013), often relying instead on the auditory modality to the exclusion of the visual information (Mongillo et al., 2008; Stevenson et al., 2014, in press). While individuals with ASD may be able to perceptually bind information under optimal conditions, these results imply that individuals with ASD show reduced efficiency when binding speech information across auditory and visual modalities, particularly in noisy, real-world contexts (Foxe et al., 2013). As a consequence, signals are perceived in isolation, or as fragmented units rather than as a meaningful whole. Thus, the efficiency gained from processing multiple sensory signals as a single percept, for example the visual sensory inputs associated with a speaker integrated with the auditory sensory inputs associated with a speaker (Stevenson et al., 2010, 2011), would be lost, resulting in more inefficient sensory processing overall.

Given the findings that individuals with ASD show reduced perceptual binding of audiovisual speech signals, it has been hypothesized that individuals with ASD would not exhibit the behavioral gains observed with the perception of multisensory signals. The few studies to date that have investigated multisensory perception of audiovisual speech have shown that children with ASD do in fact show less behavioral gain (i.e., less improved perception) with audiovisual speech than do their typically developing peers (Alcántara et al., 2004; Smith and Bennetto, 2007; Irwin et al., 2011; Foxe et al., 2013). This finding is especially salient when speech is embedded in a high degree of background noise (Foxe et al., 2013), the very condition in which (A) typically developing children show a high level of multisensory gain and (B), this multisensory integration would be most beneficial for successful speech communication. The validity of the relationship between multisensory perception and real-world communication has been demonstrated via correlations between the accurate perception of audiovisual speech and communication scores from the Autism Diagnostic Observation Schedule (Lord et al., 2000), the gold standard for diagnostic testing in ASD. Individuals who were better able to accurately perceive audiovisual speech were less impaired in terms of communicative abilities (Woynaroski et al., 2013).

Interestingly, multisensory speech integration is not a static process, but one that continues to mature and fine tune over development (Hillock et al., 2011; Hillock-Dunn and Wallace, 2012). While young children with ASD are clearly delayed in their ability to benefit from multisensory speech perception compared to their typically developing peers, there is evidence that this impairment lessens with maturation (Foxe et al., 2013). Likewise, the first study of the McGurk Effect across development showed a similar pattern, in which young children with ASD perceived the McGurk Effect much less frequently than their peers without ASD, but “caught up” later in development (Taylor et al., 2010; but see Stevenson et al., in press).

A critical question then, is what is the underlying cause of these disruptions in speech perception observed in ASD? One possibility is that individuals with ASD have impaired temporal processing abilities. One neurobiological account of ASD, the temporal binding hypothesis of autism (Brock et al., 2002) proposes just that. In terms of binding across sensory inputs, perceiving the timing of incoming sensory information is paramount to the ability to perceptually bind stimuli across sensory modalities. The temporal synchrony of such inputs is one, if not the most, salient cue that two inputs *should* be bound (Vroomen and Keetels, 2010). Previous research shows a clear pattern that individuals with ASD are significantly impaired in judging the relative timing of auditory and visual speech signals (Bebko et al., 2006; Foss-Feig et al., 2010; Kwakye et al., 2011; de Boer-Schellekens et al., 2013; Woynaroski et al., 2013; Stevenson et al., 2014), and importantly, this research also showed a direct correlation between multisensory temporal acuity and the ability to perceptually bind audiovisual speech signals in individuals with ASD (Stevenson et al., 2014).

These findings, taken in sum, suggest that deficits in binding across auditory and visual modalities in ASD may have a cascading impact on speech perception and social processing, key clinical symptoms defining ASD. In most social communicative interactions, failing to perceive the auditory and visual components of the environment can result in missing critical social cues, not to mention the content of the message being conveyed. Failing to perceive a speaker's message as a single, unified percept, essentially doubles the number of perceived inputs, resulting in an increasingly “noisy” or “intense” world—as is often described in the case of autism (Just et al., 2004; Markram et al., 2007; Rippon et al., 2007; Pouget et al., 2009).

The impact of an inability to perceptually bind across senses on other aspects of cognition has been well characterized in a patient with bilateral parietal hypoperfusion (Hamilton et al., 2006). This patient, AWF, began to perceive what he heard and what he saw as being out of sync. As a result of this atypical multisensory temporal processing, AWF was unable to perceptually bind audiovisual speech, indexed by an inability to perceive the McGurk Effect. Additionally, AWF no longer showed the typical behavioral benefits with he was shown a speakers mouth and articulatory gestures accompanying auditory speech. While the etiology of AWF's impairment is clearly distinct from ASD, the parallels in the perception of audiovisual speech are striking. Furthermore, AWF's describes coping with his asynchronous environment by limiting face-to-face conversations and looking away from the face during in-person conversations, both behaviors commonly seen in ASD. Such a coping strategy may reflect the perceived avoidance of social interactions in ASD, which may relate more to limiting the amount of perceptual noise in the environment. A similar argument has been made for self-stimulation or “stimming” behaviors commonly observed in ASD. It is possible that these repetitive movements provide a predictable and controlled sensory experience in an otherwise chaotic world (Jones et al., 2003).

While the impact that atypical sensory binding appears to have on the core symptoms associated with ASD is supported by research, the issue of how to translate these findings into clinical practice has been largely unexplored (note here that treatments commonly referred to as “sensory integration therapy” do not in fact focus on binding or integrating information across sensory modalities). Intensive Behavioral Intervention (IBI) is the evidence-based treatment of choice for ASD; however, the degree of gain made by any one child is difficult to predict. While milder autism severity, higher adaptive functioning, and higher cognitive skills are related to better outcomes, there remain unaccounted for factors which may predict which children benefit most from treatment (Flanagan et al., 2012). Given that sensory and multisensory processing are foundational to the higher-level cognitive, communicative, and social functioning that treatments aim to address, knowledge of an individual's ability to process sensory information is a critical and necessary first step to benefit maximally from intensive intervention.

These possible clinical implications are, at this stage, highly speculative. The possible upsides, however, of moving this research from the laboratory into real-world settings are significant. A clear consensus of evidence suggests that individuals with ASD process and integrate sensory information in an atypical manner, and that this is strongly linked to core impairments in communicative and social abilities. A number of research questions must be addressed in order to explore these possibilities. First, longitudinal studies of individuals with ASD need to be conducted to directly assess how speech and communication skills develop in conjunction with sensory processing, specifically binding across sensory modalities and multisensory temporal processing. Second, the mediating or moderating effect that specific sensory-processing phenotypes in ASD have on the efficacy of evidence-based treatments such as IBI is sorely needed (in addition to other variables such as IQ and gender; Wolery and Garfinkle, 2002; Rogers and Vismara, 2008). Finally, research should ultimately go beyond documenting the sensory and multisensory processing abilities of individuals with ASD and in addition, should also reveal how these abilities can be dynamically modulated. Plasticity within the relevant perceptual systems has been amply demonstrated (Fujisaki et al., 2004; Powers et al., 2009; Stevenson et al., 2013; Schlessinger et al., in press), but these findings have been not yet been applied to populations with ASD. Pursuing these and related studies has the potential to not only add to our understanding of ASD, but also, through clinical application, to improve the quality of life of individuals with ASD.

## Conflict of interest statement

The authors declare that the research was conducted in the absence of any commercial or financial relationships that could be construed as a potential conflict of interest.
